# Effects of Chronic Hydrocodone Exposure and Ceftriaxone on the Expression of Astrocytic Glutamate Transporters in Mesocorticolimbic Brain Regions of C57/BL Mice

**DOI:** 10.3390/toxics11100870

**Published:** 2023-10-20

**Authors:** Woonyen Wong, Youssef Sari

**Affiliations:** Department of Pharmacology and Experimental Therapeutics, College of Pharmacy and Pharmaceutical Sciences, The University of Toledo, Toledo, OH 43614, USA; woonyen.wong@rockets.utoledo.edu

**Keywords:** opioids, ceftriaxone, glutamate, GLT-1, xCT, kinases, drug exposure, mesocorticolimbic

## Abstract

Exposure to opioids can lead to the alteration of several neurotransmitters. Among these neurotransmitters, glutamate is thought to be involved in opioid dependence. Glutamate neurotransmission is mainly regulated by astrocytic glutamate transporters such as glutamate transporter 1 (GLT-1) and cystine/glutamate antiporter (xCT). Our laboratory has shown that exposure to lower doses of hydrocodone reduced the expression of xCT in the nucleus accumbens (NAc) and the hippocampus. In the present study, we investigated the effects of chronic exposure to hydrocodone, and tested ceftriaxone as a GLT-1 upregulator in mesocorticolimbic brain regions such as the NAc, the amygdala (AMY), and the dorsomedial prefrontal cortex (dmPFC). Eight-week-old male mice were divided into three groups: (1) the saline vehicle control group; (2) the hydrocodone group; and (3) the hydrocodone + ceftriaxone group. Mice were injected with hydrocodone (10 mg/kg, i.p.) or saline for 14 days. On day seven, the hydrocodone/ceftriaxone group was injected with ceftriaxone (200 mg/kg, i.p.) for last seven days. Chronic exposure to hydrocodone reduced the expression of GLT-1, xCT, protein kinase B (AKT), extracellular signal-regulated kinases (ERK), and c-Jun N-terminal Kinase (JNK) in NAc, AMY, and dmPFC. However, hydrocodone exposure increased the expression of G-protein-coupled metabotropic glutamate receptors (mGluR5) in the NAc, AMY, and dmPFC. Importantly, ceftriaxone treatment normalized the expression of mGluR5, GLT-1, and xCT in all these brain regions, except for xCT in the AMY. Importantly, ceftriaxone treatment attenuated hydrocodone-induced downregulation of signaling pathways such as AKT, ERK, and JNK expression in the NAc, AMY, and dmPFC. These findings demonstrate that ceftriaxone has potential therapeutic effects in reversing hydrocodone-induced downregulation of GLT-1 and xCT in selected reward brain regions, and this might be mediated through the downstream kinase signaling pathways such as AKT, ERK, and JNK.

## 1. Introduction

Opioid use disorder (OUD) has been considered as a major health issue in the United States [1]. Opioids are commonly used to treat chronic pain; however, their misuses are associated with the development of dependence, and overdose leading to deaths. There are several classes of opioids, and hydrocodone is considered a semi-synthetic opioid, which is widely used in the management of chronic pain associated with surgery procedures and musculoskeletal injuries [2]. Hydrocodone exerts its analgesic effect by activating the mu-opioid receptor, a G-protein coupled receptors (GPCR), which can inhibit the production of cyclic adenosine monophosphate (cAMP) leading to the activation of a G-protein-gated inwardly rectifying potassium channel (GIRK) [3]. Furthermore, opioids dysregulate several neurotransmitters, including glutamate [4]. Indeed, the activation of opioid receptors (mainly mu receptor) induced release of glutamate in the nucleus accumbens (NAc) core, and this effect was mainly observed in astrocytes [5], which highly express the major glutamate transporter type 1 (GLT-1) and the cystine/glutamate antiporter (xCT) [6,7,8,9].

Glutamate homeostasis is dysregulated by exposure to substances of abuse, including alcohol, nicotine, cocaine, and methamphetamine, and this effect has been associated with the downregulation of GLT-1 expression in several reward brain regions such as the NAc, dorsomedial prefrontal cortex (dmPFC), amygdala (AMY), and hippocampus [10,11,12,13,14,15]. In addition, xCT was also found to be downregulated during NAc exposure to substances of abuse such as cocaine, alcohol, and nicotine [16,17,18,19]. Importantly, chronic exposure to opioids alters glutamate transport and glutamate clearance. For example, chronic exposure to morphine downregulated several glutamate transporters, including GLT-1 [20,21]; this might be associated with an increase in extracellular glutamate concentrations in the brain [20,22]. Regarding xCT, a study from our laboratory showed that hydrocodone administered at a lower dose decreased the expression of this protein in the NAc and hippocampus in an animal model of conditioned place preference (CPP) [23]. Importantly, ceftriaxone, a beta-lactam antibiotic known to upregulate GLT-1 and xCT [9,24], was shown to attenuate the effect of hydrocodone-induced downregulation in xCT in these brain regions [23]. These studies and others clearly demonstrate that chronic exposure to drugs of abuse downregulated the expression of GLT-1 and xCT and increased extracellular glutamate concentrations in the central reward brain regions, and CEF and other beta-lactams have the potential to attenuate these effects.

In this study, we investigated the effects of chronic exposure to a higher dose of hydrocodone (10 mg/kg) on the expression of GLT-1 and xCT in certain central reward brain regions such as the NAc, AMY, and dmPFC. Importantly, we determined whether ceftriaxone treatment would normalize the expression of these glutamate transporters. We further investigated whether chronic exposure to hydrocodone affects the expression of mGluR5, and determined whether ceftriaxone would attenuate this effect. Finally, we aimed to investigate the signaling pathways involved in hydrocodone-induced changes in GLT-1 and xCT expression. We focused on the expression of signaling pathways such as ERK, JNK, and Akt since some of these kinases are suggested to be involved in the regulatory effect of ceftriaxone in GLT-1 expression in the brain [25,26,27].

## 2. Materials and Methods

### 2.1. Animal

Male C57BL/6 mice (Jackson Laboratory, 25–30 g, 8 weeks of age, *n* = 7) were used in this study. This study tested a total of 21 male mice. Mice were housed in a room that was maintained at 21 °C on a 12/12 h light/dark cycle. Mice had free access to water and food. All experimental procedures were approved by the Institutional Animal Care and Use Committee (IACUC), University of Toledo. This is in accordance with the guidelines set by the National Institutes of Health for the use of animals in research as described in the Guide for the Care and Use of Laboratory Animals under approved protocol number 400155 (2 August 2022), The University of Toledo. 

### 2.2. Drugs and Dosing

**Figure 1:**
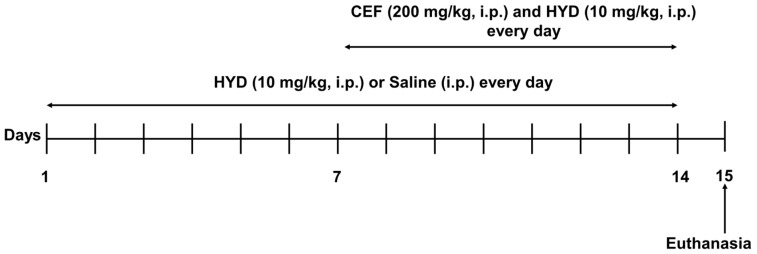
Timeline of the experimental procedure. CEF: ceftriaxone; HYD: hydrocodone.

Male mice were handled three days before starting the experiment for acclimation. Mice were then divided into three groups: (1) saline group, mice were intraperitoneal (i.p.) injected with saline vehicle from day 1–14 (*n* = 7); (2) hydrocodone group, mice were i.p. injected hydrocodone (10 mg/kg) from day 1–14 (*n* = 7); and (3) hydrocodone + ceftriaxone group, mice were i.p. injected hydrocodone (10 mg/kg) from day 1–14, and ceftriaxone (200 mg/kg) was i.p. injected from day 7–14 (*n* = 7) (Figure 1). Hydrocodone (Sigma-Aldrich, St. Louis, MO, USA) was dissolved in saline at 10 mg/kg. Ceftriaxone was purchased from (Pfizer, Lake Forest, IL, USA) and was dissolved in saline at 200 mg/kg. Note that equal volumes of saline (control group) and hydrocodone (10 mg/kg, i.p., hydrocodone group) were injected from day 1 through 14; ceftriaxone (200 mg/kg, i.p.) was administered from day 7 through 14 for the ceftriaxone/hydrocodone group. Mice were euthanized on day 15 by CO_2_ inhalation as approved by UT-IACUC (Figure 1).

### 2.3. Brain Tissue Extraction

Animals were euthanized by CO_2_ inhalation after 7 h of fasting on day 15. Fasting was applied for further study, which aims to investigate potential changes in liver tissues. For the present study, brains were isolated and frozen immediately on dry ice and stored at −80 °C. NAc (core and shell), dmPFC (cingulate cortex and prelimbic cortex), HIP (cornu ammonis, CA, subfield: CA1, CA2, and CA3), and AMY (central amygdala, basomedial amygdala and basolateral amygdala) were extracted using a cryostat machine (Leica CM1950). All brain regions were selected using the Brain Mice Atlas [28]. Brain samples were stored at −80 °C for subsequent Western blot analyses.

### 2.4. Western Blot Analysis

Western blot was used to determine protein expression of phospho-ERK, ERK, phospho-Akt, Akt, phospho-JNK, JNK, xCT, GLT-1, mGluR5, and β-tubulin in the NAc (core and shell), AMY, and dmPFC. Samples were lysed using a lysis buffer (50 mM Tris–HCl, 150 mM NaCl, 1 mM EDTA, 0.5% NP-40, 1% Triton, 0.1% SDS) with phosphatase and protease inhibitors. The amount of protein in each tissue sample was quantified using a detergent compatible protein assay (Bio-Rad, Hercules, CA, USA). An equal amount of protein from each sample was mixed with laemmili dye, and the mixtures were loaded onto 10% Tris-glycerine gel to separate the protein using electrophoresis. Then, proteins were transferred from gels to a polyvinylidene difluoride (PVDF) membrane. Subsequently, the PVDF membranes were blocked with 5% fat-free milk in Tris-buffered saline with Tween 20 (TBST) at room temperature for 30 min. Membranes were incubated overnight at 4 °C with primary antibodies: rabbit anti-*p*-ERK (1:1000, Abcam, Cambridge, UK, ab201015), rabbit anti-ERK (1:1000, Abcam, ab17942), rabbit anti-*p*-JNK (1:1000, Cell Signaling, Danvers, MA, USA, 9251), rabbit anti-JNK (1:1000, Cell Signaling, 9252), rabbit anti-*p*-Akt (1:1000, Cell Signaling, 4060), rabbit anti-Akt (1:1000, Cell Signaling, 4691), rabbit anti-GLT-1 (1:5000, Abcam ab205248), rabbit anti-xCT (1:1000, Abcam ab125186), and rabbit anti-mGluR5 (1:1000, Abcam ab76316). Mouse anti-β-tubulin (1:1000, BioLeagend, San Diego, CA, USA) was used as a control loading protein. On the following day, membranes were washed five times with TBST and incubated with the match secondary antibody (1:4000) for 60 min. The membranes were then washed with TBST and dried for further analysis. The dried membranes were incubated with chemiluminescent reagents (Super Signal West Pico, Perce Inc., Appleton, WI, USA) for 1–2 min. Digitized blot images were developed using the GeneSys imaging system. Quantification and analysis of the expression of *p*-ERK, ERK, *p*-JNK, JNK, *p*-Akt, Akt, GLT-1, xCT, mGluR5, and β-tubulin blots were performed using ImageJ software (Version 1.53t 24). The control group was reported as 100% to measure the changes in the expression of proteins of interest in the NAc (core and shell), AMY, and dmPFC as described in our previous studies [23,29].

### 2.5. Statistical Analysis

All statistical analyses were performed using GraphPad Prism software (Version 10). One-way ANOVA with Newman–Keuls as a post-hoc multiple comparison test was used to analyze the Western blot data as a percentage (relative to control values) ratio to the loading protein, β-tubulin. The data are reported for a *p* < 0.05 level of significance.

## 3. Results

### 3.1. Effect of Chronic Hydrocodone Exposure and Ceftriaxone on GLT-1 Protein Expression in the NAC, AMY, and dmPFC

**Figure 2:**
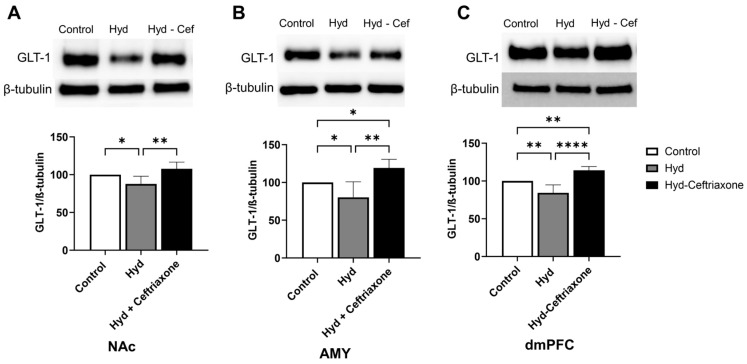
Effect of chronic hydrocodone exposure on the expression of GLT-1 in the NAc, AMY, and dmPFC. (**A**) Immunoblots for GLT-1 and β-tubulin in the NAc. Quantitative analysis using one-way ANOVA followed by Newman–Keuls post-hoc test indicated that GLT-1 was significantly downregulated in the hydrocodone group compared to the control group, while post-treatment with ceftriaxone (200 mg/kg) upregulated GLT-1 expression compared to the hydrocodone group in the NAc. (**B**) Immunoblots for GLT-1 and β-tubulin in the AMY. Quantitative analysis using one-way ANOVA followed by Newman–Keuls post-hoc test showed that GLT-1 was significantly downregulated in the hydrocodone group compared to the control group, while post-treatment with ceftriaxone (200 mg/kg) upregulated GLT-1 expression compared to the hydrocodone group in the AMY. (**C**) Immunoblots for GLT-1 and β-tubulin in the dmPFC. Quantitative analysis using one-way ANOVA followed by Newman–Keuls post-hoc test indicated that GLT-1 expression was significantly downregulated in the hydrocodone group compared to the control group, while ceftriaxone (200 mg/kg) upregulated GLT-1 expression compared to the hydrocodone group in the dmPFC. Control group data were represented as 100%. Each column is expressed as mean ± S.E.M (*n* = 7/group), (* *p* < 0.05, ** *p* < 0.01, and **** *p* < 0.0001).

Data analyses revealed a significant difference in the expression of GLT-1 in NAc among all tested groups (F_2,13_ = 11.16, *p* < 0.01, Figure 2). Newman–Keuls post-hoc analyses showed a significant decrease in GLT-1 expression in the NAc in the hydrocodone group compared to the control group (*p* < 0.05), and ceftriaxone (*p* < 0.01) significantly increased GLT-1 expression in the NAc as compared to the hydrocodone group (Figure 2A). In addition, statistical analysis revealed a significant difference in the expression of GLT-1 in the AMY (F_2,13_ = 11.16, *p* < 0.01, Figure 2B) and the dmPFC (F_2,15_ = 29.24, *p* < 0.0001, Figure 2C) among all tested groups. Newman–Keuls post-hoc analyses showed a significant decrease in GLT-1 expression in the AMY (*p* < 0.05, Figure 2B) and the dmPFC (*p* < 0.01, Figure 2C) of the hydrocodone group compared to the control group. Importantly, ceftriaxone significantly increased GLT-1 expression in the AMY (*p* < 0.01, Figure 2B) and dmPFC (*p* < 0.0001, Figure 2C) as compared to the hydrocodone group. The hydrocodone–ceftriaxone groups showed significantly increased GLT-1 expression compared to the control group in the AMY (*p* < 0.05, Figure 2B) and the dmPFC (*p* < 0.05, Figure 2C). However, no significant changes were observed between the control and hydrocodone-ceftriaxone groups in the NAc (Figure 2).

### 3.2. Effect of Chronic Hydrocodone Exposure and Ceftriaxone on xCT Protein Expression in the NAc, AMY, and dmPFC

**Figure 3:**
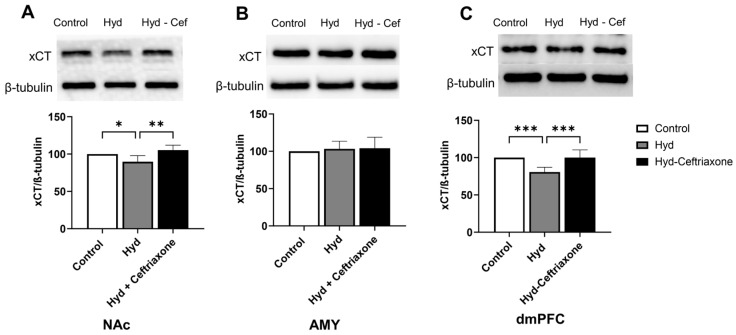
Effect of chronic hydrocodone exposure on xCT expression in the NAc, AMY, and dmPFC. (**A**) Immunoblots for xCT and β-tubulin in the NAc. Quantitative analysis using one-way ANOVA followed by Newman–Keuls post-hoc test indicated that xCT expression was significantly decreased in the hydrocodone group compared to the control group, while ceftriaxone (200 mg/kg) normalized GLT-1 expression compared to the hydrocodone group in the NAc. (**B**) Immunoblots for xCT and β-tubulin in the AMY. Quantitative analysis using one-way ANOVA followed by Newman–Keuls post-hoc test indicated that there were no significant differences in xCT expression among all tested groups in the AMY. (**C**) Immunoblots for xCT and β-tubulin in dmPFC. Quantitative analysis using one-way ANOVA followed by Newman–Keuls post-hoc test showed that xCT was significantly decreased in the hydrocodone group compared to the control group, while post-treatment with ceftriaxone (200 mg/kg) normalized xCT expression compared to the hydrocodone group in the dmPFC. Control group data were represented as 100%. Each column is expressed as mean ± S.E.M (*n* = 7/group), (* *p* < 0.05, ** *p* < 0.01 and *** *p* < 0.001).

We further investigated the effects of chronic hydrocodone exposure and ceftriaxone treatment on the expression of xCT in mesocorticolimbic brain regions. There were significant differences in xCT expression in the NAc (F_2,12_ = 8.364, *p* < 0.01, Figure 3A) and dmPFC (F_2,15_ = 15.07, *p* < 0.001, Figure 3C) among all tested groups. However, there were no significant changes in xCT expression between all tested groups in the AMY (F_2,18_ = 0.03247, *p* > 0.05, Figure 3B). Newman–Keuls post-hoc analyses revealed significant decreases in xCT expression in the NAc (*p* < 0.05, Figure 3A) and the dmPFC (*p* < 0.001, Figure 3C) in the hydrocodone group as compared to the control group. Importantly, ceftriaxone attenuated hydrocodone-induced downregulation in the NAc (*p* < 0.01, Figure 3A) and dmPFC (*p* < 0.001, Figure 3C). Quantitative analysis revealed non-significant differences in xCT expression among control and hydrocodone–ceftriaxone groups in the NAc, AMY, and dmPFC (Figure 3).

### 3.3. Effects of Chronic Hydrocodone Exposure and Ceftriaxone on p-ERK Protein Expression in the NAc, AMY, and dmPFC

**Figure 4:**
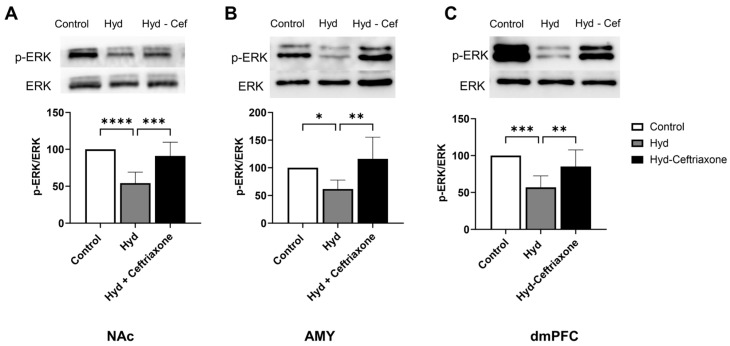
Effect of chronic hydrocodone exposure on *p*-ERK expression in the NAc, AMY, and dmPFC. (**A**) Immunoblots for *p*-ERK and ERK in the NAc. Quantitative analysis using one-way ANOVA followed by Newman–Keuls post hoc test revealed that chronic hydrocodone exposure downregulated *p*-ERK expression in the NAc compared to the control group, while ceftriaxone (200 mg/kg) upregulated *p*-ERK expression compared to the hydrocodone group. (**B**) Immunoblots for *p*-ERK and ERK in the AMY. Quantitative analysis using one-way ANOVA followed by Newman–Keuls post hoc test showed that *p*-ERK was significantly downregulated in the hydrocodone group compared to the control group, while post-treatment with ceftriaxone (200 mg/kg) upregulated *p*-ERK expression in the AMY compared to the hydrocodone group. (**C**) Immunoblots for *p*-ERK and ERK in the dmPFC. Quantitative analysis using one-way ANOVA followed by Newman–Keuls post hoc test showed that *p*-ERK expression was significantly downregulated in the hydrocodone group compared to the control group, while ceftriaxone (200 mg/kg) upregulated *p*-ERK expression in the dmPFC compared to the hydrocodone group. Control group data were represented as 100%. Each column is expressed as mean ± S.E.M (*n* = 7/group), (* *p* < 0.05, ** *p* < 0.01, *** *p* < 0.001 and **** *p* < 0.0001).

We also investigated the effects of ceftriaxone on kinase signaling pathways such as *p*-ERK in mesocorticolimbic brain regions. Western blot data analyses revealed significant differences in *p*-ERK expression in the NAc (F_2,14_ = 19.73, *p* < 0.0001, Figure 4A), AMY (F_2,13_ = 7.141, *p* < 0.01, Figure 4B), and dmPFC (F_2,14_ = 12.44, *p* < 0.001, Figure 4C) among all groups. Newman–Keuls post hoc analyses revealed that chronic hydrocodone exposure decreased *p*-ERK expression in the NAc (*p* < 0.0001, Figure 4A), AMY (*p* < 0.05, Figure 4B), and dmPFC (*p* < 0.001, Figure 4C) compared to the control group. Importantly, ceftriaxone attenuated hydrocodone-induced downregulation of *p*-ERK in the NAc (*p* < 0.001, Figure 4A), AMY (*p* < 0.01, Figure 4B) and the dmPFC (*p* < 0.01, Figure 4C). No significant changes were detected between the hydrocodone and hydrocodone–ceftriaxone groups.

### 3.4. Effect of Chronic Hydrocodone Exposure and Ceftriaxone on p-AKT Protein Expression in the NAc, AMY, and dmPFC

**Figure 5:**
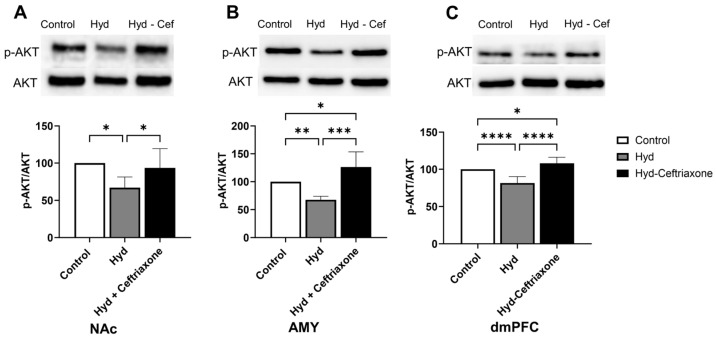
Effect of chronic hydrocodone exposure on *p*-AKT expression in the NAc, AMY, and dmPFC. (**A**) Immunoblots for *p*-AKT and AKT in the NAc. Quantitative analysis using one-way ANOVA followed by Newman–Keuls post hoc test indicated that *p*-AKT expression was significantly decreased in the hydrocodone group as compared to the control group, while ceftriaxone (200 mg/kg) normalized *p*-AKT expression in the NAc compared to the hydrocodone group. (**B**) Immunoblots for *p*-AKT and AKT in the AMY. Quantitative analysis using one-way ANOVA followed by Newman–Keuls post hoc test showed that *p*-AKT was significantly decreased in the hydrocodone group compared to the control group, while post-treatment with ceftriaxone (200 mg/kg) normalized the expression of *p*-AKT expression in the AMY compared to the hydrocodone group. (**C**) Immunoblots for *p*-AKT and AKT in the dmPFC. Quantitative analysis using one-way ANOVA followed by Newman–Keuls post hoc test showed that *p*-AKT expression was significantly reduced in the hydrocodone group compared to the control group, while ceftriaxone (200 mg/kg) normalized *p*-AKT expression in the dmPFC compared to the hydrocodone group. Control group data were represented as 100%. Each column is expressed as mean ± S.E.M (*n* = 7/group), (* *p* < 0.05, ** *p* < 0.01, *** *p* < 0.001 and **** *p* < 0.0001).

The effect of chronic hydrocodone exposure on *p*-AKT expression was also measured in mesocorticolimbic brain regions. Data analyses revealed significant differences in *p*-Akt expression in the NAc (F_2,13_ = 5.970, *p* < 0.05, Figure 5A), AMY (F_2,13_ = 18.37, *p* < 0.001, Figure 5B), and dmPFC (F_2,18_ = 27.26, *p* < 0.0001, Figure 5C) among all groups. Chronic hydrocodone exposure decreased *p*-Akt expression in the NAc (*p* < 0.05, Figure 5A), AMY (*p* < 0.01, Figure 5B), and dmPFC (*p* < 0.0001, Figure 5C) compared to the control group. Importantly, ceftriaxone attenuated hydrocodone-induced downregulation of *p*-Akt expression (*p* < 0.05, Figure 5A) in the NAc, AMY (*p* < 0.001, Figure 5B), and dmPFC (*p* < 0.0001, Figure 5C). Significant differences were observed between control and hydrocodone-ceftriaxone groups in the AMY (*p* < 0.05, Figure 5B) and dmPFC (*p* < 0.05, Figure 5C); no significant difference was detected in the NAc (Figure 5A).

### 3.5. Effect of Chronic Hydrocodone Exposure and Ceftriaxone on p-JNK Protein Expression in the NAc, AMY, and dmPFC

**Figure 6:**
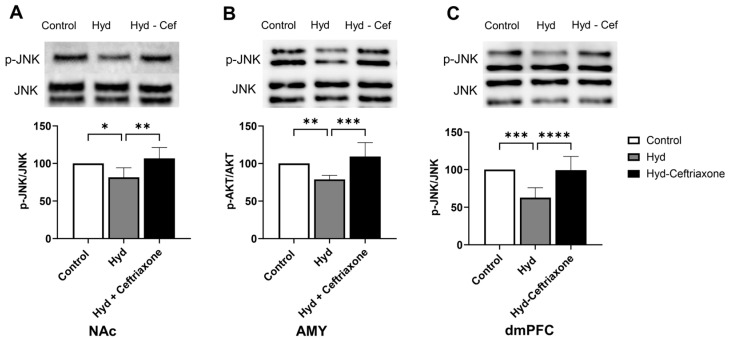
Effect of chronic hydrocodone exposure on *p*-JNK expression in the NAc, AMY, and dmPFC. (**A**) Immunoblots for *p*-JNK and JNK in the NAc. Quantitative analysis using one-way ANOVA followed by Newman–Keuls post hoc test revealed that *p*-JNK expression in the NAc was significantly downregulated in the hydrocodone group compared to the control group, while ceftriaxone (200 mg/kg) upregulated *p*-AKT expression compared to the hydrocodone group. (**B**) Immunoblots for *p*-JNK and JNK in the AMY. Quantitative analysis using one-way ANOVA followed by Newman–Keuls post hoc test showed that *p*-JNK was significantly downregulated in the hydrocodone group compared to the control group, while post-treatment with ceftriaxone (200 mg/kg) upregulated *p*-JNK expression in the AMY compared to the hydrocodone group. (**C**) Immunoblots for *p*-JNK and JNK in the dmPFC. Quantitative analysis using one-way ANOVA followed by Newman–Keuls post hoc test indicated that *p*-JNK expression was significantly downregulated in the hydrocodone group compared to the control group, while ceftriaxone (200 mg/kg) upregulated *p*-JNK expression in the dmPFC compared to the hydrocodone group. Each column is expressed as mean ± S.E.M (*n* = 7/group), (* *p* < 0.05, ** *p* < 0.01, *** *p* < 0.001 and **** *p* < 0.0001).

We further investigated the effect of chronic exposure to hydrocodone on *p*-JNK expression in mesocorticolimbic brain regions. Data analyses revealed a significant difference in *p*-JNK expression in the NAc (F_2,14_ = 7.577, *p* < 0.01, Figure 6A), AMY (F_2,15_ = 11.82, *p* < 0.001, Figure 6B), and dmPFC (F_2,18_ = 18.36, *p* < 0.0001, Figure 6C) among all tested groups. Hydrocodone exposure decreased *p*-JNK expression in the NAc (*p* < 0.05, Figure 6A), AMY (*p* < 0.01, Figure 6B), and dmPFC (*p* < 0.001, Figure 6C) compared to the control group. Importantly, ceftriaxone attenuated hydrocodone-induced downregulation in *p*-JNK expression in the NAc (*p* < 0.01, Figure 6A), AMY (*p* < 0.001, Figure 6B), and dmPFC (*p* < 0.0001, Figure 6C). No significant difference was found in *p*-JNK expression between the control and hydrocodone–ceftriaxone groups in all three brain regions (Figure 6).

### 3.6. Effect of Chronic Hydrocodone Exposure and Ceftriaxone on mGluR5 Protein Expression in the NAc, AMY, and dmPFC

**Figure 7:**
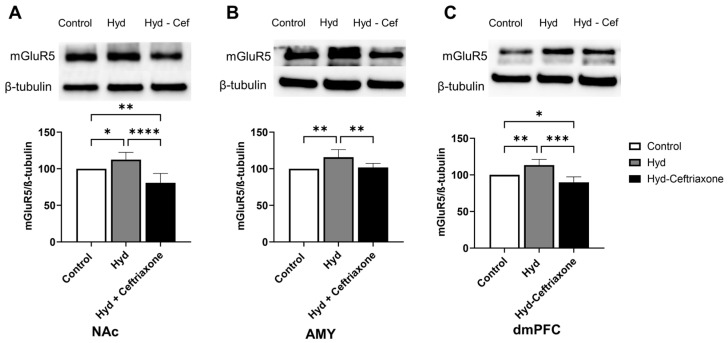
Effect of chronic hydrocodone exposure on mGluR5 expression in the NAc, AMY, and dmPFC. (**A**) Immunoblots for mGluR5 and β-tubulin in the NAc. Quantitative analysis using one-way ANOVA followed by Newman–Keuls post hoc test showed that mGluR5 expression was significantly upregulated in the hydrocodone group compared to the control group, while ceftriaxone (200 mg/kg) normalized mGluR5 expression in the NAc compared to the hydrocodone group. (**B**) Immunoblots for mGluR5 and β-tubulin in the AMY. Quantitative analysis using one-way ANOVA followed by Newman–Keuls post hoc test showed that mGluR5 was significantly upregulated in the hydrocodone group compared to the control group, while post-treatment with ceftriaxone (200 mg/kg) normalized mGluR5 expression in the AMY compared to the hydrocodone group. (**C**) Immunoblots for mGluR5 and β-tubulin in the dmPFC. Quantitative analysis using one-way ANOVA followed by Newman–Keuls post hoc test showed that mGluR5 expression was significantly upregulated in the hydrocodone group compared to the control group, while ceftriaxone (200 mg/kg) normalized mGluR5 expression in the dmPFC compared to hydrocodone group. Control group data were represented as 100%. Each column is expressed as mean ± S.E.M (*n* = 7/group), (* *p* < 0.05, ** *p* < 0.01, *** *p* < 0.001 and **** *p* < 0.0001).

We finally determined the effect of hydrocodone exposure on mGluR5 expression in mesocorticolimbic brain regions. Statistical analyses revealed a significance difference in mGluR5 expression in the NAc (F_2,15_ = 17.81, *p* < 0.001, Figure 7A), AMY (F_2,12_ = 8.018, *p* < 0.01, Figure 7B), and dmPFC (F_2,12_ = 18.59, *p* < 0.001, Figure 7C). Furthermore, chronic hydrocodone exposure increased mGluR5 expression in the NAc (*p* < 0.05, Figure 7A), AMY (*p* < 0.01, Figure 7B), and dmPFC (*p* < 0.01, Figure 7C). Importantly, ceftriaxone attenuated hydrocodone-induced upregulation in mGluR5 expression in the NAc (*p* < 0.0001, Figure 7A), AMY (*p* < 0.01, Figure 7B), and dmPFC (*p* < 0.001, Figure 7C) compared to the control and hydrocodone groups. No significant differences were observed between the control and hydrocodone–ceftriaxone groups in the AMY (Figure 7B). However, there were significant differences between the control and hydrocodone–ceftriaxone groups in the NAc (*p* < 0.01, Figure 7A) and the dmPFC (*p* < 0.05, Figure 7C).

## 4. Discussion

The hyperglutamatergic state is a major neurochemical unbalance that might be the cause of many neurological diseases and psychiatric disorders, including drug addiction [30,31]. GLT-1 plays an important role in regulating the majority of extracellular glutamate concentrations in the brain, and a reduction in GLT-1 expression is often associated with relapse to drugs of abuse. Therefore, restoring glutamate homeostasis may have a therapeutic, beneficial effect against neuroexcitotoxicity caused by chronic exposure to drugs of abuse. The present study demonstrated that chronic hydrocodone exposure alters the expression of GLT-1, xCT, mGluR5, *p*-ERK/ERK, *p*-Akt/Akt, and *p*-JNK/JNK in mesocorticolimibic brain regions. Ceftriaxone, known to upregulate GLT-1, attenuated hydrocodone-induced alteration of the expression of these target proteins. This study focused on three brain regions: the dmPFC, AMY, and NAc. These brain regions are reciprocally connecting glutamatergic projections, which are involved in drug seeking and drug dependence [6,9]. GLT-1 is a major glutamate transporter that regulates most of the extracellular glutamate concentrations in these key reward brain regions and others [8]. Previous studies from our laboratory demonstrated that chronic exposure to ethanol induced downregulation of GLT-1, as well xCT expression in central reward brain regions, including the NAc and ceftriaxone, attenuated this effect [6,16,32]. It is important to note that xCT is colocalized with GLT-1 in astrocytes to regulate basal extracellular glutamate concentrations [7]. Thus, both GLT-1 and xCT are critical in regulating the excess of extracellular glutamate that is mediated through astrocytes. In this study, we focused on the effect of chronic exposure of hydrocodone in the mesocorticolimbic brain regions involved drug dependence.

Exposure to hydrocodone (10 mg/kg, i.p.) for 14 days downregulated GLT-1 expression in the dmPFC, AMY, and NAc. In contrast to our current findings, previous studies from our laboratory reported no change in GLT-1 expression with hydrocodone exposure (5 mg/kg, i.p.) in the NAc, dmPFC, hippocampus, and AMY [23]. However, this study showed that hydrocodone exposure downregulated xCT expression in the NAc and the hippocampus, and ceftriaxone attenuated this effect. This difference may be attributable to the duration of hydrocodone exposure and the dose of hydrocodone tested. In addition, it has been suggested that a reduction in GLT-1 expression in the NAc is associated with chronic exposure to drugs of abuse, including alcohol, nicotine, heroin, and amphetamine [12,16,33,34]. Hence, our study showed that chronic exposure to higher doses of hydrocodone resulted in a decrease in GLT-1 expression in the dmPFC, AMY, and NAc. Furthermore, we found that xCT expression was downregulated in the dmPFC and NAc, but not in the AMY. The result from this current study is in accordance with our previous study which showed that exposure to a lower dose of hydrocodone (5 mg/kg, i.p.) in alcohol-preferring (P) rats reduced xCT expression in the NAc in a CPP model [23]. In the present study, ceftriaxone restored GLT-1 expression in the brains of mice exposed to hydrocodone. We suggest that the upregulatory effect of ceftriaxone in GLT-1 expression may decrease extracellular glutamate concentrations and increase glutamate uptake in models of drugs abuse, including ethanol and opioids [6,24]. Treatment with ceftriaxone reversed the effects of hydrocodone-induced downregulation of GLT-1 and xCT expression in the dmPFC and NAc. This is consistent with previous findings indicating the downregulation of GLT-1 and xCT expression in the NAc of animals exposed to drugs of abuse, including hydrocodone, ethanol, cocaine, nicotine, and methamphetamine, and these involved different behavioral paradigms such as drug seeking, self-administration, and reinstatement [11,15,23,35,36,37]. Our present findings demonstrated clearly that β-lactams (e.g., ceftriaxone and other β-lactams) have the potential to attenuate the effects of chronic exposure to opioids and normalize glutamate transporters to prevent neuroexcitotoxicity that might be mediated through excess of extracellular glutamate concentrations at the synaptic cleft.

**Figure 8:**
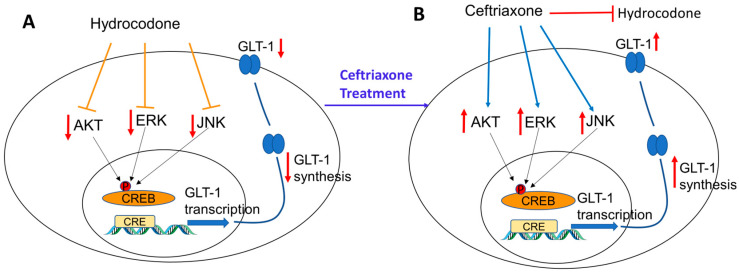
Schematic representation summarizing the effects of chronic hydrocodone exposure on GLT-1 expression in mesocorticolimbic brain regions. (**A**) Chronic exposure to hydrocodone reduced ERK, AKT, and JNK signaling kinases leading to GLT-1 downregulation. (**B**) Ceftriaxone treatment attenuated hydrocodone-induced GLT-1 downregulation by upregulating signaling kinases such as ERK, AKT, and JNK.

Furthermore, this study explored the signaling pathways involved in GLT-1 and xCT upregulation. Thus, we focused on investigating the effect of hydrocodone exposure in Akt expression and its phosphorylated form. The Akt pathway is involved in synaptic and structural neuroadaptations, and *p*-Akt expression was decreased in the NAc after exposure to drug abuse, including morphine, heroin, and nicotine [38,39,40]. These findings are in accordance with our present findings showing that *p*-Akt is downregulated in the dmPFC, AMY, and NAc after chronic exposure to hydrocodone. Several studies reported that the Akt signaling pathway, which functions downstream of phosphatidylinositol 3-kinase (PI-3K) and the nuclear transcription factor-κB (NF-κB), is involved in the upregulation of GLT-1 expression [41]. Additionally, our studies and others confirmed the association between the Akt pathway and the upregulation of GLT-1 expression [41,42,43,44]. Our results showed that *p*-Akt expression was upregulated after ceftriaxone treatment, suggesting that the Akt signaling pathway is involved in a ceftriaxone-mediated increase in GLT-1 (Figure 8).

We further investigated the ERK signaling pathway as it is involved in neuroplasticity, and signal transduction [45]. Our study showed that exposure to hydrocodone for 14 days downregulated *p*-ERK expression in the dmPFC, AMY, and NAc, and this effect was attenuated with ceftriaxone treatment. Downregulation of ERK in the NAc is in accordance with previous studies showing that chronic morphine exposure reduced both ERK and Akt in the NAc of male Sprague-Dawley rats and CD-1 mice [38,46,47]. In addition, *p*-ERK is also known to initiate the transcription factors such as NF-kB and CREB, which in turn regulate GLT-1 transcription [48]. Therefore, our current study further showed that ceftriaxone activated the ERK signaling pathway, and consequently modulated GLT-1 expression (Figure 8).

Furthermore, we investigated the JNK pathway as an important signaling pathway in the mitogen-activated protein kinases (MAPK) family. A study has reported that CREB activation is dependent on JNK [49]. Additionally, CREB phosphorylation is required for synaptic plasticity and memory consolidation [50,51,52]. In this study, we showed that chronic exposure to hydrocodone reduced *p*-JNK expression in the dmPFC, AMY, and NAC. Hydrocodone-induced downregulation of *p*-JNK expression was attenuated after ceftriaxone treatment. Here, we suggest that upregulation of GLT-1 expression in the dmPFC, AMY, and NAc might be associated with activation of CREB through JNK phosphorylation [53] (Figure 8). However, studies are warranted to validate this assumption.

In addition to signaling pathways, mGluRs have also been implicated in opioid reward. mGluR5 is known to play a facilitative role in mediating the potentiating effects of opioids and is highly expressed in reward-related brain regions, including the NAc and dmPFC [54]. It is important to note that mGluR5 is selectively increased in the NAc under a morphine-CPP paradigm and repeated exposure to cocaine [55,56]. This is in accordance with our present study demonstrating that chronic exposure to hydrocodone increased mGluR5 expression in the dmPFC, AMY, and NAc. Hydrocodone-induced upregulation in mGluR5 expression was attenuated with ceftriaxone treatment.

## 5. Conclusions

This study revealed that chronic exposure to hydrocodone induced dysregulation of glutamatergic system in the NAc, AMY, and dmPFC. Treatment with ceftriaxone successfully attenuated hydrocodone-induced dysfunction in this glutamatergic system. This was associated with the reversal of hydrocodone-induced changes in mGluR5, GLT-1, xCT, ERK, AKT, and JNK expression in the NAc, AMY, and dmPFC. We revealed that the upregulatory or normalizing effect of ceftriaxone in GLT-1 expression was mediated in part through the kinase signaling pathways such as the ERK, AKT, and JNK.

This study was proof of a concept to determine the effects of exposure to hydrocodone for 14 days on the expression of astrocytic glutamate transporters (GLT-1 and xCT). The study was limited to determine the signaling pathways involved in the upregulatory effects of ceftriaxone on GLT-1 and xCT expression. Further studies are warranted to investigate the beneficial preclinical effects of ceftriaxone and other beta-lactams in a model of self-administration or CPP of hydrocodone and other highly potent opioids (e.g., fentanyl). In addition, further studies are warranted to investigate the beneficial preclinical effects of ceftriaxone-induced upregulation of GLT-1 and xCT on a model of opioid overdose.

## Data Availability

The data presented in this study are available in this research article.
